# Characterization of the complete mitochondrial genome of the Yunnan endemic grasshopper *Longchuanacris curvifurculus* (Insecta: Orthoptera: Catantopidae)

**DOI:** 10.1080/23802359.2018.1481777

**Published:** 2018-06-11

**Authors:** Zhou Hu, Yu-Peng Han, De-Long Guan, Ben-Yong Mao

**Affiliations:** a College of Agriculture and Life Sciences, Dali University, Dali, Yunnan, China;; b College of Pharmacy and Chemistry, Dali University, Dali, Yunnan, China;; c College of Life Sciences, Shaanxi Normal University, Xi’an, Shaanxi, China

**Keywords:** Assembly by reduced complexity (ARC), Illumina sequencing, mitochondrial genome, *Longchuanacris curvifurculus*

## Abstract

*Longchuanacris curvifurculus* (*L. curvifurculus*) was once a dominating grasshopper in the Yunnan province (People’s Republic of China) that occupy important ecological niche. However, its population has severely declined because of the deterioration of ecological environment. Identifying the species and source of *L. curvifurculus* is important for biodiversity conservation and ecological/environmental preservation. In the study, the complete mitochondrial genome of *L. curvifurculus* was assembled from high-coverage (36.8×) Illumina MiSeq sequencing data. The circular genome is 15,450 bp in length, harboring 37 typical mitochondrial genes and one control region. The nucleotide composition is asymmetric (43.0% A, 14.3% C, 10.5% G, and 32.2% T), with an overall A + T content of 75.2%. All the protein-coding genes (PCGs) are initiated with typical ATN start codons and terminated by the typical TAA codons or the incomplete T(aa) codon. The control region has a remarkably high A + T content (84.9%) and is located between genes *rrnS and trnV*.

*Longchuanacris curvifurculus* (*L. curvifurculus*) is a type of short-horned grasshopper belonging to the order Orthoptera and family Catantopidae (Ramme 2008). Systematic studies of *L. curvifurculus* that lead to more effective and precise identification of this species, and will be important for biodiversity conservation and monitoring as well as for the management and control of population levels. Mitochondrial DNA, a powerful molecular tool for genetic research, has proven to be useful for such purposes (Song 2010; Zhao et al. 2010). In this investigation, the complete mitochondrial genome of *L. curvifurculus* has been assembled from whole-genome sequencing data generated using the Illumina MiSeq sequencing system (Illumina, San Diego, CA). The annotated mitogenomic sequence has been deposited into GenBank under the accession number MF989227.

An individual of *L. curvifurculus* was used in this study and the type sample of this species is now persevered in Museum of Da’li University, Yunnan, China. In all, 24.141 million raw reads were retrieved, quality-trimmed with Trimmomatic v0.35 (Bolger et al. 2014), and then used for the assembly of the mitochondrial genome with the Assembly by Reduced Complexity (ARC) pipeline (http://ibest.github.io/ARC/) (Hunter et al. 2015). This pipeline implements a hybrid mapping and assembly approach for targeted assembly of homologous sequences. A mitogenomic fragment (GenBank: EF437157) was used as the initial reference. About 3850 individual mitochondrial reads generated an average coverage of 36.8. Genome annotation was conducted using the MITOS Web Server (http://mitos.bioinf.uni-leipzig.de/index.py) (Bernt et al. 2013; Jansen et al. 2005; Papadakos et al. 2008), with precise adjustment via alignment with other available Catantopidae mitochondrial genomes (Liu and Qiu 2016; Yang et al. 2016).

The mitochondrial genome of *L. curvifurculus* is 15,450 bp in size, with a highly asymmetric nucleotide composition (43.0% A, 14.3% C, 10.5% G, and 32.2% T) and an overall A + T content of 75.2%. It harbours 13 protein-coding genes (PCGs), 22 tRNA genes, two rRNA genes (*rrnS* and *rrnL*), and one control region (Figure 1). All 13 PCGs are initiated at typical ATN start codons, and are terminated with a TAA codon except *nad5* and *nad2* with the incomplete T(aa) codons. Sizes of the 22 tRNAs vary over a very small range, from 63 bp (*trnC*) to 71 bp (*trnK* and *trnV*). The two adjacent rRNAs are 1276 bp (*rrnL*) and 781 bp (*rrnS*) in length, with A + T contents of 77.60% and 74.20%, respectively. The control region, which is located between *rrnS* and *trnV*, is 617 bp long and has a remark-ably high A + T content (84.90%).The arrangement of genes is identical to that of other Catantopidae mitochondrial genomes (Hu et al. 2017).

**Figure 1.**
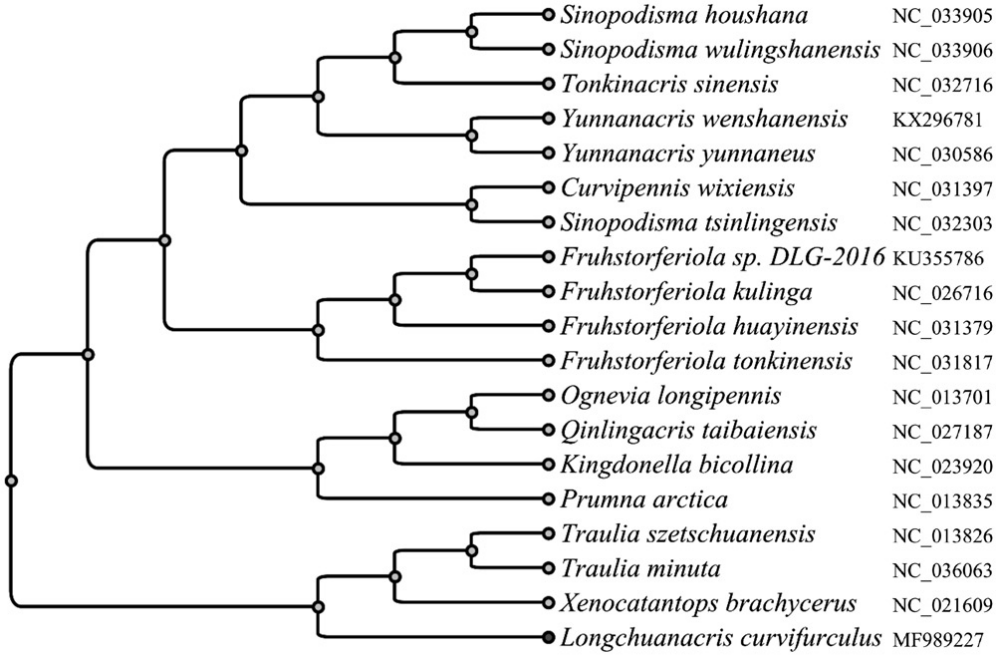
Phylogenetic analysis using the 13 mitochondrial protein-coding genes of *Longchuanacris curvifurculus*. Mitochondrial genomes with the genbank accession ID of NC_033905, NC_033906, NC_032716, KX296781, NC_030586, NC_031397, NC_032303, KU355786, NC_026716, NC_031379, NC_031817, NC_013701, NC_027187, NC_023920, NC_013835, NC_013826, NC_036063, NC_021609, along with our data MF989227, were used to build the phylogenetic tree.

Using the sequences of 13 PCGs from the obtained mitochondrial genome of *L. curvifurculus* and 19 other Catantopidae/Melanoplinae mitochondrial genomes, an UPGMA tree was built to reveal the phylogenetic relationship among these species (Figure 1). The results have shown that *L. curvifurculus* is close to genus Traulia and Xenocatantops, which are in consist with the previous morphology classifications.
